# Supplementary figures and images for: Combining Short- and Long-Read Sequencing Technologies to Identify SARS-CoV-2 Variants in Wastewater
Source: Viruses. 2024 Sep 21;16(9):1495. doi: 10.3390/v16091495 (PMC11437403; doi:10.3390/v16091495)

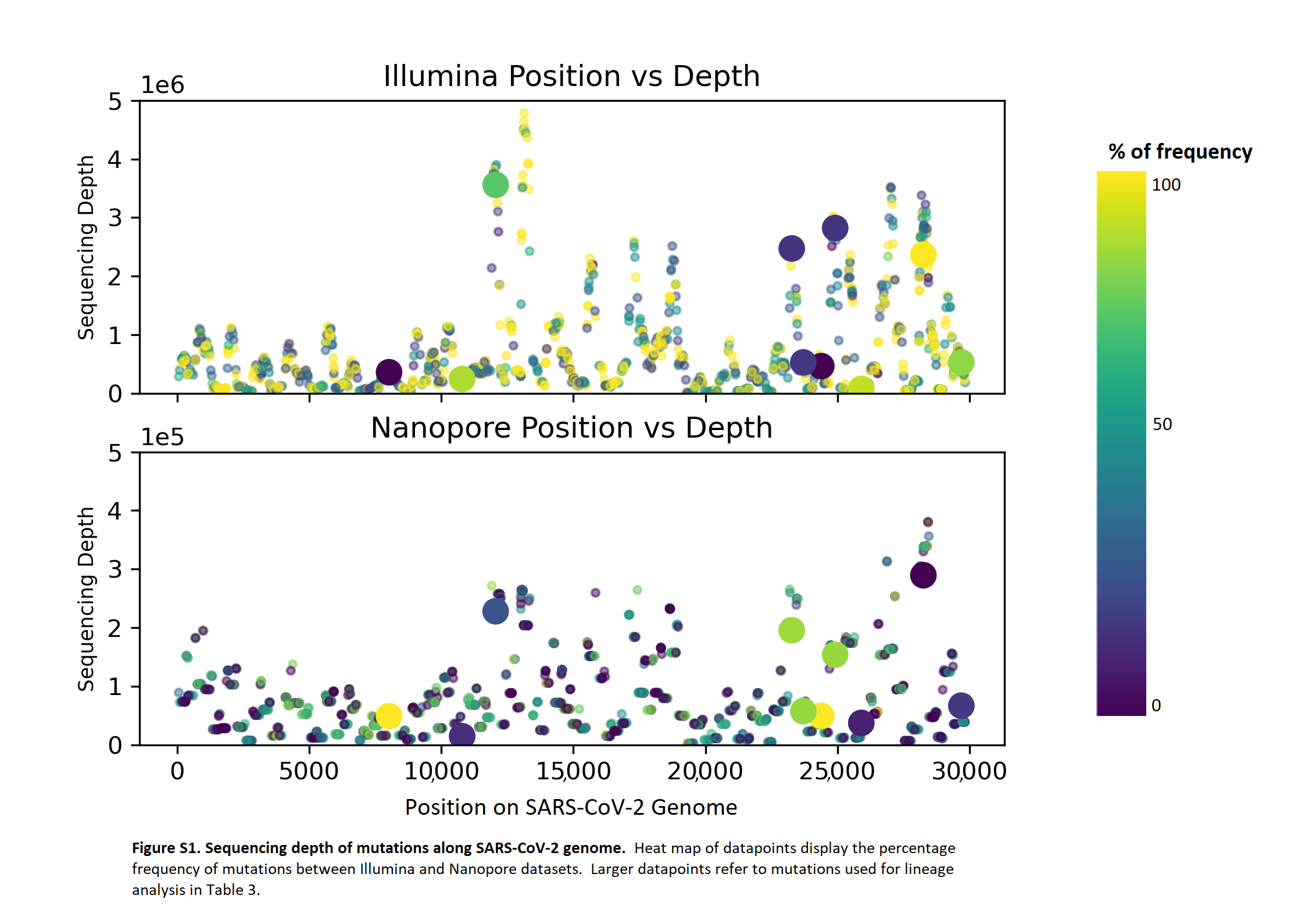

Supplement: Supplementary file 1 [file viruses-16-01495-s001.zip › Figure_S1_Sequencing_depth_of_mutations_along_SARS-CoV-2_genome.png]

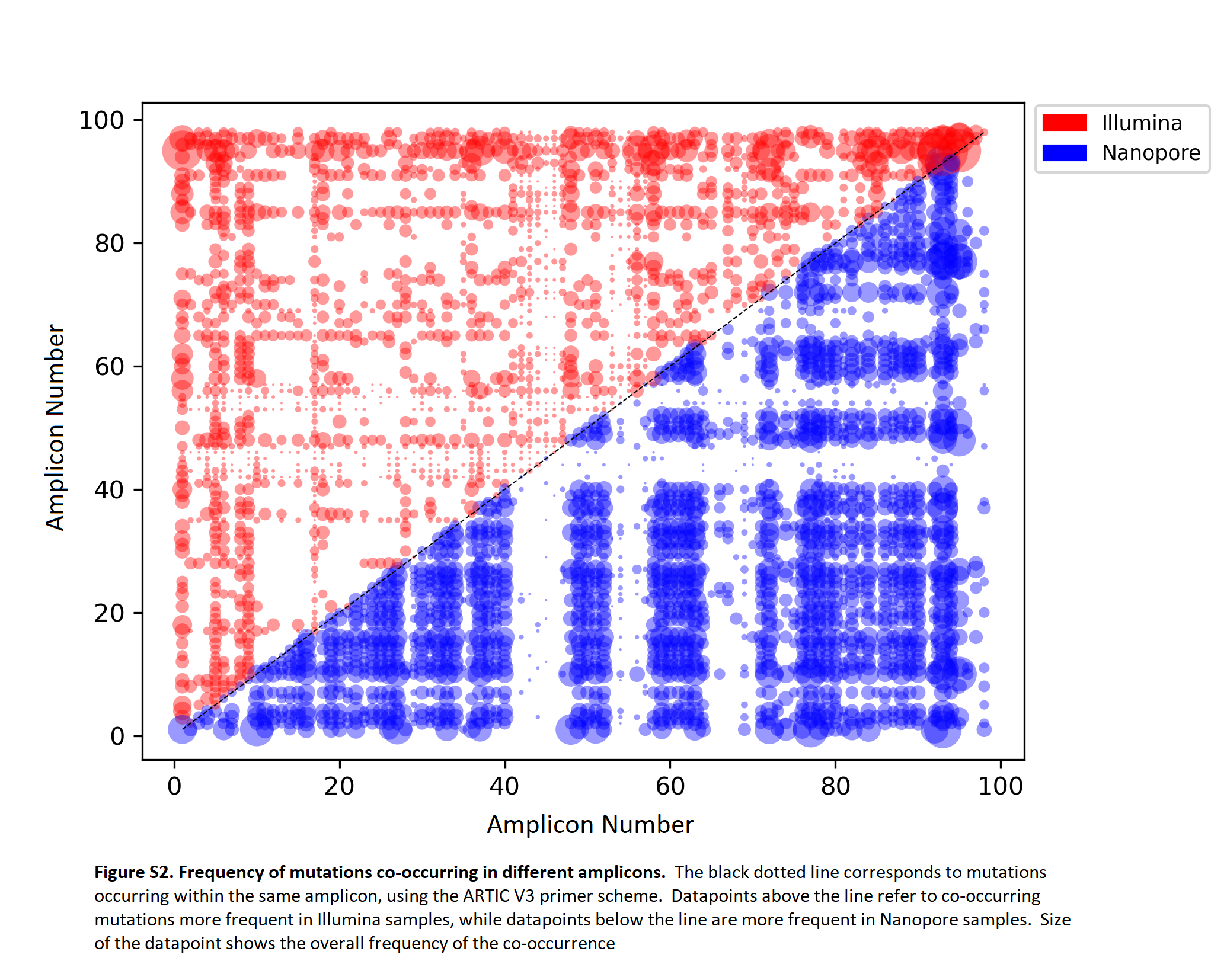

Supplement: Supplementary file 1 [file viruses-16-01495-s001.zip › Figure_S2_Frequency_of_mutations_co-occurring_in_different_amplicons.png]
